# Patient and Provider Experiences With Compassionate Care in Virtual Physiatry: Qualitative Study

**DOI:** 10.2196/51878

**Published:** 2024-08-06

**Authors:** Marina B Wasilewski, Abirami Vijayakumar, Zara Szigeti, Amanda Mayo, Laura Desveaux, James Shaw, Sander L Hitzig, Robert Simpson

**Affiliations:** 1 Sunnybrook Research Institute St. John's Rehab Toronto, ON Canada; 2 Institute for Better Health Mississauga, ON Canada; 3 University of Toronto Toronto, ON Canada

**Keywords:** compassionate care, rehabilitation, physiatry, telemedicine, burnout, care, qualitative study, patient experience, compassion, compassionate, patient centered, virtual care, communication, health care delivery, patient engagement, physiatrist, physiatrists, Canada, social media, physical medicine, technology, communication technology, ICT, experience

## Abstract

**Background:**

Telemedicine in the realm of rehabilitation includes the remote delivery of rehabilitation services using communication technologies (eg, telephone, emails, and video). The widespread application of virtual care grants a suitable time to explore the intersection of compassion and telemedicine, especially due to the impact of COVID-19 and how it greatly influenced the delivery of health care universally.

**Objective:**

The purpose of this study was to explore how compassionate care is understood and experienced by physiatrists and patients engaged in telemedicine.

**Methods:**

We used a qualitative descriptive approach to conduct interviews with patients and physiatrists between June 2021 and March 2022. Patients were recruited across Canada from social media and from a single hospital network in Toronto, Ontario. Physiatrists were recruited across Canada through social media and the Canadian Association for Physical Medicine and Rehabilitation (CAPM&R) email listserve. Interviews were recorded and transcribed. Data were analyzed thematically.

**Results:**

A total of 19 participants were interviewed—8 physiatrists and 11 patients. Two themes capturing physiatrists’ and patients’ experiences with delivering and receiving compassionate care, especially in the context of virtual care were identified: (1) compassionate care is inherently rooted in health care providers’ inner intentions and are, therefore, expressed as caring behaviors and (2) virtual elements impact the delivery and receipt of compassionate care.

**Conclusions:**

Compassionate care stemmed from physiatrists’ caring attitudes which then manifest as caring behaviors. In turn, these caring attitudes and behaviors enable individualized care and the establishment of a safe space for patients. Moreover, the virtual care modality both positively and negatively influenced how compassion is enacted by physiatrists and received by patients. Notably, there was large ambiguity around the norms and etiquette surrounding virtual care. Nonetheless, the flexibility and person-centeredness of virtual care cause it to be useful in health care settings.

## Introduction

The COVID-19 pandemic has profoundly impacted health care delivery around the world. Like many health disciplines [1,2], physical medicine and rehabilitation (PM&R) (also known as “physiatry” or “rehabilitation medicine”) pivoted to “virtual care”—or “telemedicine”—to mitigate the spread of COVID-19 and to ensure that patients had continued access to vital rehabilitation services throughout the pandemic [3,4]. “Telemedicine” in the context of rehabilitation involves the remote provision of rehabilitation services using information and communication technologies either synchronously (eg, phone calls and videoconferences) or asynchronously (eg, email) to improve patient health [5,6]. Telemedicine models enabled physiatrists (PM&R physicians) to continue providing care to patients, to promote functional recovery and improve quality of life [7]. An abundance of evidence across typical PM&R patient populations (eg, stroke, chronic musculoskeletal conditions, and chronic obstructive pulmonary disorder) has demonstrated telemedicine to be as effective as in-person care at improving several physical (eg, motor function) and social (eg, quality of life) health outcomes [8-12]. Despite these clinical and practical advantages, the implementation of telemedicine was limited prior to the pandemic [13,14], when it was driven forward by necessity at a pace that left little room to optimize its delivery.

Telemedicine introduces the potential to disrupt the relational nature of the traditional doctor-patient relationship, including complex, relational processes such as empathic connection, rapport building, and compassionate responding, which form the basis of compassionate care [15]. Compassion itself is a multifaceted concept that can be broadly defined as the emotions that arise when noticing someone suffer, and feeling motivated to help reduce such suffering through relational care and action [16]. Viewed widely as a core aspect of high-quality health care, compassionate patient care entails human-to-human “contact,” personal interaction, and bidirectional communication [17]. Many of these implicit processes that take place during in-person encounters may not be the same in virtual care contexts. For example, health care workers have expressed that many long hours of providing care virtually leads to exhaustion, due to a heightened need to concentrate on the screen and overcompensate for the challenges associated with processing nonverbal cues (eg, body language, facial expressions) [18]. This potentially puts providers at higher risk for burnout and compassion fatigue [18], especially concerning given that physiatrists are the third most burnt out medical specialty in North America [19].

The widespread implementation of virtual care presents an opportune time to explore the emerging intersection of compassion and telemedicine. A recent scoping review on the conceptualization, use, and outcomes associated with compassion in PM&R found that no studies had explored the concept of compassion in a virtual care setting [20]. What compassionate care means to health care providers and patients in PM&R settings and how it is conveyed and experienced virtually represents a key knowledge gap. The aim of this study was to explore how compassionate care is understood and experienced by physiatrists and patients engaged in telemedicine. Although there are a range of theoretical approaches to answer research questions, many designs may not be optimal for studies that do not necessitate a highly theoretical framework and instead focus closely on capturing and understanding participants’ experiences [21]. Hence, a qualitative descriptive approach may be used to provide experiences and perceptions of study participants [22] to better understand the conceptualization of compassionate care among patients and health care providers.

## Methods

### Research Design

We used a qualitative descriptive approach, which entails a concise and descriptively rich analysis that remains true to participants’ own words. Thus, it produces a data-near report that is representative of participants’ views, making it meaningful for key stakeholders and relevant for justifying actionable change [22,23].

### Participants

#### Participant Profile

Eight Physiatrists and 11 patients across Canada were recruited for this study.

#### Inclusion and Exclusion Criteria

Participants were eligible to participate if they were English speaking adults and judged able to provide informed consent. Patient participants had to have received care virtually, at least once, from a physiatrist in the 90 days preceding recruitment. Physiatrist had to have provided virtual care at least once in the previous 90 days. Virtual care included video, telephone, and email consultation or follow-up visits (or both).

#### Recruitment Procedure

Between June 2021 and March 2022, we recruited physiatrists across Canada via social media and the Canadian Association for Physical Medicine and Rehabilitation (CAPM&R) email listserve. Patients were recruited across Canada from social media and [REHABILITATION HOSPITAL—DE-IDENTIFIED] outpatient clinic via referral by a member of their circle of care.

### Ethical Considerations

This study was approved by the Research Ethics Board at Sunnybrook Hospital (ID: 4960). Informed consent was obtained prior to data collection.

### Data Collection

Once informed consent was collected for participants, one-on-one interviews were conducted by two trained qualitative researchers (AV and ZS) via telephone or Zoom (Zoom Video Communications) between June 2021 and March 2022. Interview questions were focused on participants’ experiences with providing and receiving virtual care and the ways that compassion featured in these interactions (see Multimedia Appendices 1 and 2 for interview questions). Data were collected until saturation of ideas was achieved (ie, no new information emerged from subsequent interviews) [24]. The participants had no prior relationship with the interviewers and understood that the study goals were to explore experiences with virtual care. Data sharing is not applicable to this article as no data sets were generated or analyzed during this study.

Interviews ranged from 25 to 75 minutes, were audio-recorded, and transcribed verbatim. All identifying information was removed from the transcripts, which were uploaded to NVivo [25] for organization and analysis. Sociodemographic information was collected from both patients and physiatrists—for the latter we also collected professional practice information (eg, years of practice, practice setting).

### Data Analysis

We used an inductive thematic approach following the steps outlined by Braun and Clarke (2006), whereby data were deconstructed into isolated fragments, followed by reconstruction into overarching themes which describe the higher-level messaging in the data [26]. Two independent researchers (AV and ZS) completed the coding process and 2 additional researchers (RS and MBW) participated in the thematic analysis.

### Rigor

Analytic rigor was enhanced by triangulating between multiple individuals throughout analysis, having regular team meetings, and practicing reflexivity (discussing and journaling the study team’s own biases and experiences that may influence data interpretation) [24]. We also adhered to the consolidated criteria for reporting qualitative research (COREQ) guidelines (See Appendix A). The COREQ checklist is used to encourage explicit and clear documentation of interview methodologies, ensuring important details are included in this document. Refer to Domains 2 and 3 of the COREQ to see how study design, analysis and findings were outlined. Furthermore, a generative AI was not used in any portion of the manuscript writing.

## Results

We interviewed n=19 participants (n=11 patients and n=8 physiatrists; see Table 1 for participant characteristics). We identified 2 key themes that captured patients’ and physiatrists’ experiences with receiving and providing compassionate care, broadly, and their experiences within the virtual context, specifically.

**Table 1:** Demographic information for patient and health care professional participants (n=19).

Characteristics			Patients (n=11)		Health care professionals (n=8)
Age (years), mean (SD)			62.2 (9.5)		42.8 (8.5)
**Assigned sex at birth, n (%)**					
	Male	4 (36)		4 (50)	
	Female	7 (64)		4 (50)	
**Gender identity, n (%)**					
	Male	4 (36)		4 (50)	
	Female	7 (64)		4 (50)	
**Ethnicity, n (%)**					
	White-European	7 (64)		3 (37.5)	
	White-North American	3 (27)		3 (37.5)	
	Asian-East	1 (9)		2 (25)	
**Reason for psychiatric care, n (%)**					
	Amputation	7 (64)		—^a^	
	Stroke	3 (27)		—	
	Bypass surgery	1 (9)		—	
**Received virtual care prior to the COVID-19 pandemic?, n (%)**					
	Yes	8 (73)		—	
	No	3 (27)		—	
**Primary areas of practice, n (%)**					
	Traumatic brain injury	—		1 (12.5)	
	Spinal cord injury	—		3 (37.5)	
	Stroke/neurology	—		5 (62.5)	
	Musculoskeletal	—		3 (37.5)	
	Neuromuscular/electromyography	—		2 (25)	
	Geriatric	—		1 (12.5)	
	Burns	—		1 (12.5)	
Years practicing in physiatry, mean (SD)			—		8.1 (6.8)
**Primary practice settings, n (%)**					
	Acute care facility	—		2 (25)	
	Specialist inpatient rehabilitation hospital	—		8 (100)	
	Long-term care or other congregate care setting	—		1 (12.5)	
	Other: outreach clinics	—		2 (25)	

^a^Not applicable.

### Theme #1: Compassionate Care Is Rooted in Providers’ Inner Caring Intentions that Are Expressed as Outward Caring Behaviors

Our findings suggest that compassionate care (or lack thereof) transcended health care discipline and was understood as stemming from caring attitudes which then manifested as caring behaviors. Caring attitudes and behaviors were enacted to create a safe environment, where patients felt comfortable in discussing their health concerns. Physiatrists were, thus, able to appreciate patients’ perspectives, which facilitated individualization of care.

### Caring Attitudes Manifest as Caring Behaviors

Compassionate behaviors demonstrated by health care professionals stemmed from their compassionate attitudes and intentions. When patients talked about caring attitudes, they explained that their physiatrists were being “nice” and “pleasant” (Patient 10), “kind” (health care provider [HCP] 08), as well as exercising “patience” (Patient 09). Additionally, physiatrists commonly reported being “sympathetic and empathetic” (HCP05, HCP08) to better understand the needs of their patients and respond in a “respectful and humane” (HCP01) manner.

While having kindhearted intent was highlighted as a foundation for physiatrists to exercise compassion, caring attitudes were not sufficient in isolation. Rather, participants noted that compassionate intentions needed to be expressed in the form of caring behaviors to “show me [my physiatrist] cares” (Patient 03). For example, patients noted that caring attitudes, such as “empathy” (Patient 01, Patient 03, Patient 05, Patient 09, HCP02, HCP03, HCP05, HCP08), were reflected in attentive behaviors like “actively listening about the situation” (Patient 01). In one instance, a patient described how a physiatrist “respond[ed] to me within ten minutes of my email and getting me an appointment quickly”, reinforcing that the patient's concerns were going to be “nipped in the bud” (Patient 03). Patients also felt empathy from their physiatrists when they “spent time with you and talking to you [...], that I think there’s an element of understanding, some compassion” (Patient 05).

Physiatrists’ caring behaviors were often focused on cultivating interpersonal connections and expressing interest in and respect for patients’ concerns and values—understanding that “there’s a person there [...] it’s not just the body, it’s the mind, and how that will affect [the patient] in the future” (Patient 02). Caring behaviors were rooted in “recognizing that this is a person and the person is experiencing suffering” (HCP04); hence, a compassionate approach involved alleviating this suffering rather than just “making a diagnosis and giving somebody a medication” (HCP04). Patients additionally described caring behaviors as humanizing, such as when physiatrists referred to “patients as people and using their name” (Patient 08) and when there was “the caring feeling that you get [from] somebody when you talk to them, you know they’re really interested or they really care. It’s almost like they become your friend” (Patient 05). Patients often described the importance of feeling “that interconnectedness,” which was facilitated by physiatrists “asking how I’m feeling” (Patient 09) or “call[ing] back to things about [patients’] lives that they’ve mentioned … because [the patient] knows that I’ve been listening” (HCP03). Interconnectedness was described as being facilitated by “being present and listening and taking no notes” (HCP01), “offer[ing] an assuring pat on the arm” and “reassuring them that they have my undivided attention. I’ll say a statement like, I’m here for you. How can I help you?” (HCP07).

At times, caring behaviors were clinically oriented like exercising therapeutic touch or “going out of their way” (Patient 06) to provide care, such as in the case of Patient 06’s physiatrist who was “teaching other doctors how to save limbs. And, he's also going out to the Indigenous community and doing the services for them. So, when you hear somebody doing that, then you know that they care.”

### Having Caring Attitudes and Behaviors Facilitates the Creation of a Safe Space for Individualized Care

Through a combination of caring attitudes and behaviors, patients felt that physiatrists created a safe and comfortable zone where psychological stressors were reduced, and space was created to ask questions and express concerns and fears while their physiatrist remained “non-judgmental” (Patient 08). A safe space was shaped by “giving the time for me” (Patient 02, Patient 03, Patient 10); time for physiatrists “to listen to me, hear me out […] compassionate care to me is just accepting me as I am […] not making me feel judged or less than […] that I can be myself” (Patient 03). Participants noted that a safe space was created over a prolonged period characterized by their physiatrist “know[ing] me” (Patient 04) because physiatrists “really develop[ed] rapport over a longer period of time” (HCP05) with their patients, making them feel safe and “comfortable” (Patient 04, Patient 08) enough to “start rattling off question after question […] and it’s like, perfect. She knows me, she knows that I want to try to answer questions and that she immediately feels comfortable” (HCP05). Some patients also spoke about the importance of feeling “wanted” (Patient 08), which stemmed from physiatrists being attentive and allowing them to “ramble on and communicate because I was coming to terms with certain thoughts [...] she allowed for [some self-discovery]” (Patient 01). Physiatrists described the skills needed to create a safe environment, where “listening is a big part of providing an atmosphere that feels open, supportive, so that patients feel like they can open up a fair bit and say what is actually on their mind” (HCP05).

When patients were able to express their questions, concerns, and needs in a safe, nonjudgmental space, physiatrists were better able to individualize care. Patients felt that care was individualized when physiatrists remembered “who I am and that you know me… you don't have to flip through your notes to find out who the heck I am” (Patient 03). Patients noted that their physiatrist knowing who they were, individualizing their care, and “understand[ing] what’s best for the individual” (Patient 01) occurred when physiatrist were able to “identify that not everyone’s the same and use their professional experience to alter that message to be more specific to what they hear from a condition standpoint. I feel that’s compassion” (Patient 01). Conversely, patients felt that the antithesis to individualized care was a “cookie-cutter approach” (Patient 01) that left patients feeling that their physiatrists were “putting [them] in one of the buckets” (Patient 01) without acknowledging their individual histories, limitations, and goals. Physiatrists described individualizing care by incorporating patients’ interests and meaningful activities into care plans, which was referred to as “personalizing the experience” (HCP08). For example, “let’s say, somewhere in the interview, they mention that they love to walk. And they can’t walk because of their foot drop, I will try to work that into conversation later” (HCP01). Physiatrists emphasized that “putting myself in [the patient’s] shoes” (HCP06) allowed them to better understand and acknowledge patients’ unique interests and circumstances (eg, availability of family support).

### Compassionate Care Is Absent When Caring Attitudes and Behaviors Are Lacking

Patients referenced encounters with health care providers more broadly to describe that uncaring attitudes manifested as providers “being rude” (Patient 02, Patient 05, Patient 07), “not car[ing] about [losing my leg]” (Patient 10) and being unsympathetic toward patient limitations, which hindered the provision of compassionate care. For some, descriptions of uncompassionate HCPs included individuals who were “excellent doctors but crappy human beings [that] had no bedside manners” (Patient 03). For others, this meant “a gruff attitude…and the feeling that they don’t give a damn” (Patient 05). Patients described an absence of compassionate care when their providers exhibited uncaring behaviors such as “rushing” (Patient 02, Patient 06) them and having “one-sided discussions'' (Patient 01) where patients were not given the opportunity to discuss their concerns. In some cases, the absence of caring attitudes was attributable to a lack of time. For instance, one patient said “I have found [physiatrist] to be quite compassionate when she’s not too busy. If she’s really busy and time is short, I would say she’s very matter of fact as opposed to being compassionate” (Patient 05).

### Theme #2: Virtual Elements Influence the Provision and Receipt of Compassionate Care

Participants highlighted many unique advantages to virtual care including that “you can pretty much do it anywhere” (HCP02) and provide it to “anyone” (HCP01), thereby making care “more efficient, time-wise” (HCP04). Despite the fact that virtual care “expanded a lot of opportunity” (HCP02) to access care, participants also highlighted challenges associated with virtual care, such as technical glitches or the fact that not everyone “knew how to do [virtual care]” (HCP03).

### The Flexibility of Virtual Care Modalities Allows for Individualized Compassionate Care

The ability to “[give] patients a choice” (HCP01, HCP06, HCP07) of virtual modality (eg, phone, videoconferencing, and email) and platform (eg, Zoom and Doxy.me) was viewed across participants as being compassionate as it promoted a sense of “flexibility” (Patient 03, Patient 10, HCP01), “ease” (Patient 08), “comfort” (HCP01, HCP04, HCP05, Patient 06, Patient 08, Patient 09), and autonomy. When options were provided, patients felt like their physiatrists were being “more personal, more private and more one on one” (Patient 03). Physiatrists explained that “when the [patient] actually felt like [the virtual visit] was more convenient for them” it resulted in a “really meaningful visit” and that “giving [patients] that choice” is what they “enjoy the most” (HCP02, HCP07). Providing virtual care options, specifically those with visual features (eg, Zoom and Doxy.me), was also described as advantageous for individualizing care since physiatrists could “get a sneak peek into their [patient’s] home life” (HCP02) and see elements of a patient that perhaps could not be seen otherwise. For example, physiatrists mentioned how they have “had patients show me them dancing with their grandchildren in the backyard on their cell phone […] or having them get up and show me something from their house that wouldn’t necessarily be available if they were just in a clinic, [that] personalis[es] the experience [...] And that really is helpful on virtual care because you get those intimate moments, even though you’re distanced” (HCP08). Furthermore, the unique features and flexibility of virtual modalities allowed for better family engagement in care appointments because “[patients] can have a family member with them, which often times in the pandemic or even before the pandemic, they couldn’t if they had come into clinic physically” (HCP05). Ultimately, this facilitated care individualization because families could offer supplemental information that physiatrists could then use to better-tailor care to the patient.

### The Ambiguous Nature of Virtual Care Norms Altered How Caring Attitudes and Behaviors Were Enacted

#### Ambiguity Concerning Safety

Physiatrists emphasized that the highest priorities of medicine were to avoid patient harm and ensure patient safety. This norm was still noted in a virtual world, where physiatrists described safety concerns that prevented them from “examin[ing] [the patient]” because they were in an inappropriate or unsafe environment, like “sitting in a truck at the side of the road on a construction site” (HCP03). Physiatrists noted how taking appointments from inappropriate locations was “a bit of a challenge because some patients don’t treat it like a medical appointment … they’re distracted” (HCP01). Patients agreed that they did not always take their medical appointments seriously and that they were “a little bit too comfortable because […] I didn’t have to get dressed up, I didn’t have to make any kind of an impression” (Patient 08), illustrating uncertainty as to what was and was not appropriate etiquette for virtual appointments.

Physiatrists also said that, because of the types of populations that they worked with (eg, people with complex disabilities, impaired mobility, high falls risks), they “have to be cognisant of certain safety aspects of what's happening with the patient. If they're not steady, you can't get them up to stand because they might fall” (HCP02). In addition, the ambiguity around ensuring patient safety in a virtual setting was described in relation to concerns around “consent, confidentiality, and documentation…It was like…we need to do virtual care yesterday. Here’s what you [need] to do [to] make sure you’ve covered your bases” (HCP07). Overall, physiatrists’ comments highlighted that physical safety and privacy were of paramount importance. This meant first ensuring the protection of patients’ health information and offering virtual care options that were used “in a safe way” (HCP03)—a way that considered both confidentiality and patient choice because “as long as patients provide the level of consent, you can use any virtual platform that you wish” (HCP03).

#### Ambiguity Concerning Presence

Participants’ perspectives varied when it came to norms around facilitating a sense of presence and feeling as if their physiatrist was in the moment with them. Patients noted how certain behaviors, like a physiatrist “tak[ing] notes while they were typing into a computer,” left patients feeling like their physiatrist was not really present and “not really engaged with me” (Patient 09). Some patients highlighted how feeling connected to their physiatrist was particularly difficult in an online setting because of uncaring behaviors like when “there’s one eye always on the clock to make sure that you can make the next meeting. I just found that you get that impression in any video meeting, but I don’t get that as much in the in-person meetings” (Patient 07). Physiatrists agreed that “it’s difficult to engage in the same way” (HCP02) because interpersonal caring behaviors that help facilitate a person-to-person connection are often intangible and can be challenging to enact in an online setting. Regardless, it was universally understood that enacting interpersonal caring behaviors and facilitating a sense of engagement were easier “when you already have a relationship with the patient” (HCP07) because “virtually it’s really difficult to create that connection […] that interconnection is just not being established as well. That rapport is just not happening” (Patient 09).

There were also varied comments around promptness and timing in a virtual setting. In some cases, physiatrists expressed being “more comfortable” if “a phone [appointment] ends up being 15 or 20 minutes late, because [the patient] is on the phone … they’re not sitting in clinic, doing nothing but wait[ing] for me” (HCP01). Conversely, other physiatrists felt worried that if they were late to a virtual appointment, patients may “sign off or get cut off” (HCP02). The prevailing sentiment among physiatrists, however, was that regardless of the modality, being “on time is very good” (Patient 07) because a lack of punctuality was viewed as cold, disrespectful of patient’s time, and inherently “uncaring.”

### Compassionate Behaviors and Individualized Care Are Challenged in Virtual Physiatry

Virtual care challenged physiatry practice both in terms of exercising core clinical tasks that required physical presence, and in exercising compassion. Physiatrists strongly emphasized that being physically present with patients was particularly important for physiatry because a “physical examination is very much a standard part of a physiatry assessment. And it's important for multiple reasons in terms of assessing function […] thinking about things like dexterity, things like mobility […] things that [have] to be assessed hands on” (HCP04). Patients echoed similar sentiments explaining that “if [PHYSIATRIST] wanted to look at my leg [to help with the prosthetic] […] well you can talk about it over the phone, but you can’t fix a prosthetic unless you go in” (Patient 07). The inability to perform clinical tasks and physically assess a patient further challenged physiatrists in getting “a sense of who the person is, what their needs are” (HCP01), which made it difficult for them to individualize and tailor care. Physiatrists also noted that other clinical behaviors like “handing [the patient] a tissue if they start to cry” (HCP02) or “putting your hand on their shoulder. […] even maybe hugging a patient” (HCP05) were tested when there was physical separation. The inability to enact many caring behaviors in the absence of physical presence left physiatrists feeling like they did not always “know the patient” or “know what [they] can do to support them” (HCP05). Since the virtual environment precludes many typical caring behaviors, patients stated that their physiatrists needed to “compensate for the fact you’re not physically there. The fact that you can’t tap me on the arm or you can’t rub my arm and say you’re going to be fine if I start to cry, I think you need something else to make me feel that you’re listening” (Patient 09).

## Discussion

### Principal Findings

This qualitative descriptive study explored the experiences of patients and physiatrists engaged in virtual care with the goal of understanding how compassion was expressed and received in the virtual modality. First, findings underscored that compassionate care stemmed from providers’ caring attitudes, which manifested as caring behaviors. Participants felt compassionate care was characterized by the creation of a psychologically safe space for patients where care could be individualized. Second, findings suggest that the virtual modality both positively and negatively impacts how compassion is enacted by physiatrists and received by patients. There was also ambiguity around the norms and etiquette for virtual care.

The way that compassionate care was described in our study aligns with the growing body of literature on this topic. Participants emphasized that compassion began with providers’ internal motivations and benevolent intentions but that those intentions necessarily had to manifest as tangible behaviors and expressions of care. This echoes other research that has demonstrated that while “virtuous intent” is a main driver of compassion, compassionate care is cultivated through “contemplative practices” (eg, actions, behaviors, self-reflection) [27]. In essence, providers’ internal feelings, values, and morals are what drive actions that aim to alleviate pain and suffering in others, which is the heart of compassionate care [28]. These findings suggest that providers’ intentions and motivations positively impact the care received by patients. Many participants described compassionate care in terms of the psychologically safe environment that it enabled. This included statements about feeling “safe” to ask questions and express concerns, knowing that their physiatrist knew them “as a person” (eg, recalling details about their life and family without checking notes), and feeling that their physiatrist gave them “time” (ie, not rushing through appointments). In accordance to previous studies, creating a “safe space” and making patients feeling valued, important, and “known” are all important outcomes of compassionate acts by providers [29,30]. Hence, these results support the idea that compassionate care often is received by patients in a psychologically safe location.

Conversely, participants in our study felt that compassion was lacking when providers’ behaviors and acts did not reflect concern or care for the patient (eg, provider being rude, rushing through an appointment, or not listening to the patient). This highlights that even though these providers may have had good intentions, patients will not experience compassionate care unless those intentions translate into caring acts and behaviors. Even though individual providers’ “baseline compassion aptitude” varies, skills in compassionate responding can be taught, for example, learning to enhance affective and relational components through role playing [31]. Indeed, while arguments have been made in the past that compassionate is innate and that you either “have it or you do not,” mounting research supports the notion that compassion can be cultivated through learned behaviors, principles, and strategies [32-34]. These findings raise intriguing questions regarding the nature of compassion and how it is learned and expressed by providers.

The virtual modality was viewed as impacting *expressions* of compassion by providers and *receipt* of compassion by patients. A notable theme here was that virtual care’s naissance underpinned many ambiguities about the appropriate norms and etiquette within this setting. In the most extreme cases, physiatrists expressed concerns about their patients engaging in virtual appointments from inappropriate, potentially unsafe locations, which undermined compassionate care in as far as physiatrists were primarily concerned with ensuring their patient’s safety. This issue has been noted in other literature and points to the challenge of providers having limited control over a patient’s virtual care environment and etiquette [35]. Some recommendations to address this issue include providers communicating expectations to patients around how to safely and optimally engage in virtual care appointments [35] and providing education that helps patients value virtual care in the same way as in-person care [36].

On the provider side, the idea of “webside” manner has gained a lot of traction with the rapid pivot to virtual care throughout the pandemic and refers to “virtual care etiquette” to be exercised by providers [37]. Good “webside” manner entails noting patient needs, taking time to clarify any concerns, and following up with their care as a way of exercising kindness and compassion in the virtual setting [37]. In our study, a lack of “webside manner” was experienced by some patients who felt that an interpersonal connection with their provider was absent and that their provider was not always “present” and “in the moment” (eg, taking notes on screen, looking off at a clock). One element that was ubiquitously mentioned by participants as mitigating this challenge was having an established patient-physiatrist relationship that could then translate to the virtual setting. This aligns with other studies of compassionate care that have emphasized the importance of mutual trust and continuity of care between patients and providers [38]. In the absence of a pre-existing relationship, providers must be additionally intentional in building rapport with patients and working to make appointments feel less rushed and more conversational [38].

Our focus on physiatry elucidated that virtual care can be especially challenging for the field of PM&R due to physiatrists’ scope of practice and the core clinical tasks in which they routinely engage. In this context, physical examination is a central part of standard practice (eg, physical manipulation of limbs, assessing prosthetics, assessing function and dexterity, etc). Although telemedicine can be optimally used for symptom monitoring, prescribing, medical advice, and psychological support (ie, tasks that typically do not require any physical presence [39]), other rehabilitation interventions (eg, physical assessments and examinations) do not necessarily have good validity when conducted virtually compared to in person [40,41]. This emphasizes that in the field of PM&R especially, there may be many opportunities to leverage the advantages of virtual care, but that in-person assessment and care remain necessary under certain conditions.

Although the virtual setting posed several challenges to compassionate care, it is important to note that the flexibility afforded by the modality was unanimously considered as an enabler of compassionate and person-centered care by patients and physiatrists. Person-centered care is defined as care that respects an individual’s preferences, needs, and values, and is provided in an empathic and compassionate way [42]. In alignment with this definition, participants in our study felt that the flexibility afforded by virtual care led to care being more individualized, considerate of unique needs and circumstances. Moving forward—during and beyond the COVID-19 pandemic—it is important to build on the many advantages of virtual care that have been identified, particularly in terms of enhancing accessibility to diverse patient populations, while also mitigating less favorable aspects of the virtual environment which otherwise might serve to undermine the provision of compassionate patient care.

### Strengths and Limitations

To the best of our knowledge, this is the first study to focus on compassionate care in the field of physiatry and in the context of virtual care. We were able to achieve a more complete understanding of compassionate care by including both physiatrist and patient perspectives. This allowed us to elucidate how compassion is both *enacted* and *experienced* in physiatry practice broadly, and in the virtual care context more specifically. One limitation is that nearly all participants were Caucasian and lived or practiced in major metropolitan areas, making our findings less transferable to patients and providers of other ethnicities and those who live or practice in rural settings. Our study also took place during a period where compensation mechanisms for virtual care where enhanced and streamlined due to the COVID-19 pandemic, which eased provision of this type of care and allowed for exploration of compassion in this context. Thus, our findings may not be transferable to circumstances when these mechanisms are no longer in place (due to reduction or elimination of virtual care compensation codes).

### Conclusions

Although virtual care has the potential to optimize patients’ access to therapy and treatment, great caution must be taken to maintain the compassionate elements of care; especially in the field of PM&R. Compassion is rooted in providers’ inner caring attitudes which manifest as caring behaviors and enable individualized care and the creation of a safe space for patients. Notably, the physical nature of core clinical tasks in physiatry was challenged by the virtual setting. Moving forward, the flexibility and person-centeredness of virtual care render it a useful modality in a routine sense, as long as in-person care is provided too, when necessary. Greater patient education, clinical guidance, and competency training for physiatrists in this area may help optimize safe and compassionate virtual care in PM&R settings.

## Appendix

Multimedia Appendix 1 Patient Interview Guide.

Multimedia Appendix 2 Physiatrist Interview Guide.

## Abbreviations

CAPM&R: Canadian Association for Physical Medicine and Rehabilitation
COREQ: consolidated criteria for reporting qualitative research
HCP: health care provider
PM&R: physical medicine and rehabilitation
