# Supplementary material for: Patient and Provider Experiences With Compassionate Care in Virtual Physiatry: Qualitative Study
Source: J Med Internet Res. 2024 Aug 6;26:e51878. doi: 10.2196/51878 (PMC11336505; doi:10.2196/51878)
Supplement: Multimedia Appendix 1 [file jmir_v26i1e51878_app1.docx]

**Multimedia Appendix 1: Patient Interview Guide**

Thank you for agreeing to participate in an interview. We have provided the interview guide so that you know the type of questions to expect and to enable you to think about your responses prior to the interview. For this interview, we are hoping to gain a better understanding of your experience with receiving rehabilitation using virtual modalities (e.g. video, phone, text). We are also interested in learning about your experience with receiving compassionate care in the context of telerehab. Compassionate care may mean different things to different people and we hope to learn what you understand about it and how you have (or have not) experienced it while receiving telerehab.

***The following is a list of events/occurrences that require you to stop the interview. In addition to this list, use your discretion in assessing the circumstances that might indicate that a participant is feeling too pressured, uncomfortable or upset to continue the interview.***

1. ***If a participant explicitly says to stop the interview***
2. ***If the participant verbally or physically implies that they would like the interview to stop (e.g. participant says “I don’t think I can do this”, etc…)***
3. ***If the participant is crying to the point of being unable to speak***

***Upon stopping, ask the participant if they would like to reschedule the rest of the interview for a later date, if they feel they can continue after taking a short break, or if they would like to drop-out of the study.***

***Contextual Information***

1. Can you please start by telling me a little bit about yourself and why you are receiving care at St. John’s Rehab?
   1. What journey brought you to St. John’s Rehab? (illness, injury, combination of the two)
   2. What type(s) of services have you received at St. John’s Rehab? (either in-person prior to pandemic or virtually during the pandemic)

***Focus on Telerehab***

1. How have you found virtual rehab compared to in-person rehab?
   1. What do you like? What do you dislike?
   2. What should be maintained?
   3. What could be improved?
2. What has largely remained the same? What do you feel is different?
3. How have your virtual interactions with your physiatrist compared to your in-person ones?

***Focus on Compassionate Care***

1. Could you tell me what you understand compassion to mean?
   1. Can you give me an example of an experience you’ve had (either in your work or personal life) that captures what being compassionate means to you
2. What does compassion in the context of healthcare mean to you?
3. How important is it to you to receive compassion from your physiatrist?
4. How does your physiatrist get to know you as a person during telerehab appointments?
5. How does your physiatrist respond to the worries and concerns you voice during telerehab appointments?
6. How does your physiatrist make you feel when they are caring for you over phone/text/video?
   1. Can you give me an example of a telerehab appointment where you felt well-cared for by your physiatrist?
7. Do you feel that your physiatrist has been compassionate during telerehab appointments? Please explain.
   1. Can you give an example of when your physiatrist has been compassionate?
   2. Can you give an example of when you felt your physiatrist was not compassionate enough?
8. How do you think physiatrists could show you more compassion during telerehab appointments?
   1. What could they say? What could they do?

We’ve discussed a number of topics and ideas today. Do you have any final thoughts or things you’d like to add to our conversation?

Thank you for taking the time to share your experiences and insights.

Before we wrap up, we will ask you some brief demographic questions. I will write these answers down in an encrypted word document to ensure your data is protected.

1. **Assigned Sex at Birth:** Male  Female Prefer Not to Answer
2. **Current Gender Identity: How do you describe yourself?**

Female  Male Transgender Female  Transgender Male

Genderqueer/gender non-conforming

Other Identity (Please Specify):____  Prefer not to answer

1. **Age:** ­­­____Years
2. **Ethnicity/background:** Asian – East (e.g., Chinese, Japanese, Korean)  Asian – South (e.g., Indian, Pakistani, Sri Lankan)  Asian – South East (e.g., Malaysian, Filipino, Vietnamese)  Black – African (e.g., Ghanaian, Kenyan, Somali)  Black – Caribbean (e.g., Barbadian, Jamaican)  Black – North American (e.g., Canadian, American)  First Nations  Indian – Caribbean (e.g., Guyanese with origins in India  Indigenous/Aboriginal – *not included elsewhere*  Inuit  Latin American (e.g., Argentinean, Chilean, Salvadoran)  Métis  Middle Eastern (e.g. Egyptian, Iranian, Lebanese)  White – European (e.g., English, Italian, Portuguese, Russian)  White – North American (e.g., Canadian, American)  Mixed Heritage (e.g., Black – African & White – North American) Please specify _____________  Other(s): Please specify _____________  Do not know  Prefer not to answer
3. **First three characters of your postal code: __ __ __**
4. **Reason for physiatry care? ______________________________________________________**
5. **Prior to the COVID pandemic, had you received virtual care from your physiatrist (i.e. via email, text, phone, or video chat?)** Yes  No  Prefer Not to Answer
