# Supplementary material for: Patient and Provider Experiences With Compassionate Care in Virtual Physiatry: Qualitative Study
Source: J Med Internet Res. 2024 Aug 6;26:e51878. doi: 10.2196/51878 (PMC11336505; doi:10.2196/51878)
Supplement: Multimedia Appendix 2 [file jmir_v26i1e51878_app2.docx]

**Multimedia Appendix 2: Physiatrist Interview Guide**

Thank you for agreeing to participate in an interview. We have provided the interview guide so that you know the type of questions to expect and to enable you to think about your responses prior to the interview. For this interview, we are hoping to gain a better understanding of your experience with providing rehabilitation to patients using virtual modalities (e.g. video, phone, text). We are also interested in learning about your experience with providing compassionate care to patients in the context of telerehab. Compassionate care may mean different things to different people and we hope to learn what you understand about it and how you have (or have not) been able to exercise it while providing telerehab.

Generally, compassionate care in a healthcare context comprises four key elements:

- A relationship based on empathy, emotional support and efforts to understand and relieve a person’s distress, suffering or concerns;
- Effective interactions between participants over time and across settings;
- Staff, patients and families being active participants in decision making; and
- Contextualised knowledge of the patient and family both individually and as members of a network of relationships.

***The following is a list of events/occurrences that require you to stop the interview. In addition to this list, use your discretion in assessing the circumstances that might indicate that a participant is feeling too pressured, uncomfortable or upset to continue the interview.***

1. ***If a participant explicitly says to stop the interview***
2. ***If the participant verbally or physically implies that they would like the interview to stop (e.g. participant says “I don’t think I can do this”, etc…)***
3. ***If the participant is crying to the point of being unable to speak***

***Upon stopping, ask the participant if they would like to reschedule the rest of the interview for a later date, if they feel they can continue after taking a short break, or if they would like to drop-out of the study.***

***To begin, I’d like to learn a little about you and your work.***

1. Can you please start by telling me where you work and what your role entails?
   1. What is your role? How long have you been in this role? What populations do you work with?
   2. What does your practice typically entail? For example, what does a typical day look like?

***I’d like to now focus on your experiences with providing virtual care to physiatry patients.***

1. Can you tell me a bit about your experience with providing virtual care?
   1. When did you start providing virtual care (during or prior to COVID-19 pandemic)?
   2. How comfortable do you feel providing virtual care?
   3. What modalities do you use? (phone, videochat, etc) Do you have a preference of modality?
   4. What have you enjoyed?
   5. What has been challenging?
   6. Broadly speaking, what could be done to improve your experience?
2. How do you feel virtual care compares to in person care?
   1. What is it like interacting with patients virtually vs. in person?
   2. In what ways do your roles and responsibilities change or remain the same when providing virtual care (compared to in-person care)?
3. What do you do in between virtual care appointments to maintain your well-being?

***I’d like to now focus a little bit on the concept of ‘compassion’, and specifically, ‘compassionate care’.***

1. To start, could you tell me what you believe ‘compassion’ and ‘compassionate care’ mean?
   1. For example, how would you describe ‘compassionate care’ to someone?
   2. What elements/characteristics of ‘compassionate care’ are important to you?
   3. What does ‘compassion’ look like in practice? Can you give me an example of an experience you’ve had (either in your work or personal life) that captures what being compassionate means to you?
2. How (if at all) does ‘compassion’ look different in a virtual care setting? How does this compare to in person care?
   1. Can you give me an example of an experience you’ve had while providing virtual care that captures what being compassionate means to you?
   2. Which virtual modalities are most compatible with promoting compassionate care?
3. What has made it easier for you to exercise compassion towards patients during virtual care appointments?
   1. How can this be maintained? What types of supports would you need? (e.g. education, professional development, peer support, organizational support, time off, etc)
4. What has made it challenging or hard to exercise compassion towards patients during virtual care appointments?
   1. How can this be mitigated? What types of supports would you need? (e.g. education, professional development, peer support, organizational support, time off, etc)

We’ve discussed a number of topics and ideas today. Do you have any final thoughts or things you’d like to add to our conversation?

Thank you for taking the time to share your experiences and insights.
